# Modified hydrologic regime of upper Ganga basin induced by natural and anthropogenic stressors

**DOI:** 10.1038/s41598-021-98827-7

**Published:** 2021-09-30

**Authors:** Somil Swarnkar, Pradeep Mujumdar, Rajiv Sinha

**Affiliations:** 1 Interdisciplinary Centre for Water and Research (ICWaR), Indian Institute of Science (IISc), Bangalore, India; 2 Department of Earth Sciences, Indian Institute of Technology Kanpur (IITK), Kanpur, India

**Keywords:** Hydrology, Environmental impact

## Abstract

Climate change and anthropogenic activities pose serious threats to river basin hydrology worldwide. The Ganga basin is home to around half a billion people and has been significantly impacted by hydrological alterations in the last few decades. The increasing high-intensity rainfall events often create flash flooding events. Such events are frequently reported in mountainous and alluvial plains of the Ganga basin, putting the entire basin under severe flood risk. Further, increasing human interventions through hydraulic structures in the upstream reaches significantly alter the flows during the pre-and post-monsoon periods. Here, we explore the hydrological implications of increasing reservoir-induced and climate-related stressors in the Upper Ganga Basin (UGB), India. Flow/sediment duration curves and flood frequency analysis have been used to assess pre-and post-1995 hydrological behaviour. Our results indicate that low and moderate flows have been significantly altered, and the flood peaks have been attenuated by the operation of hydraulic structures in the Bhagirathi (western subbasin). The Alaknanda (eastern subbasin) has experienced an increase in extreme rainfall and flows post-1995. The downstream reaches experience reservoir-induced moderate flow alterations during pre-and post-monsoon and increasing extreme flood magnitudes during monsoon. Furthermore, substantial siltation upstream of the reservoirs has disrupted the upstream–downstream geomorphologic linkages.

## Introduction

Since 1901 the global average surface temperature has risen by 0.89° due to direct and indirect impacts caused by human activities on earth system processes^1,2^. In turn, global warming has significantly impacted the local and regional hydrological cycle worldwide^3–5^. Significant variability in rainfall frequency and magnitude due to changing hydrometeorological conditions has been reported across the globe^6–9^. As a result, severe flooding to drought conditions have become more frequent and have significantly impacted socio-economic activities in different parts of the world^10^. In addition, direct human activities such as changes in land cover, surface & groundwater withdrawal, and operations of hydraulic structures have also significantly altered the river basin hydrology in several regions^11,12^.

In general, dams and reservoirs play a significant role in attenuating flood peaks, frequency, duration and magnitude globally, particularly low flows^13,14^. Further, dams and reservoirs have also disrupted the sediment delivery to the downstream reaches, causing alteration in river channel morphology and downstream sediment starvation^15,16^. Consequently, river deltas are sinking at unprecedented rates worldwide^17^. Apart from the hydrological, ecological, and societal stresses caused by these large dams and reservoirs, previous researchers have also questioned their economic viability^18,19^.

In India, rivers largely govern freshwater resources and are considered as the lifeline of the nation. More than 70% of the rural population depends upon freshwater resources for irrigation and agricultural demands fulfilled by several large and small rivers in India^20^. However, it has been observed that the changing climatic conditions in recent decades have significantly increased the extremity of severe droughts and devastating flooding events in several parts of the country^21–23^. The Himalayan regions are one of the worst affected regions in the recent decades^24–27^. Further, several major Himalayan Rivers, particularly the Ganga River basin, are regulated by more than 300 hydraulic structures (planned, commissioned and under construction) to harness the hydropower and cater for agricultural water demands^28,29^. As a result, the upstream–downstream linkages of hydrological, geomorphological and ecological processes in the Himalayan River basins are severely impacted^30–34^. Therefore, river practitioners and scientists need to understand the implications of hydrological modifications caused by changing climate and anthropogenic activities.

In the Himalayan regions of the Ganga basin, several studies have been done to assess the impacts of land use land cover change^35,36^, sediment dynamics^37–39^, climate change hazards^24–26,40^, heavy metal^41^, water quality^42^ and glacier meltwater contribution^43^. Nevertheless, detailed studies focusing on hydrological alterations caused by these hydrological structures and changing climatic conditions are currently lacking. Therefore, in this work, we select the Upper Ganga Basin (UGB) to assess the role of changing climatic conditions and increasing human activities on stream flows. The available hydrological dataset at different gauging stations was used to perform the hydrological analysis for pre-and post-dam conditions in the UGB. We first estimate the changing magnitudes of flows at those locations where large hydraulic structures were built before 1995 in the UGB. We further investigate the hydrological changes owing to changing climatic conditions and operations of hydraulic structures. Finally, we have assessed how these upstream hydrological modifications altered the hydrological regimes of the downstream regions of the UGB. The inferences drawn from the present study would be immensely useful for sustainable river basin management.

## Study area

**Figure 1:**
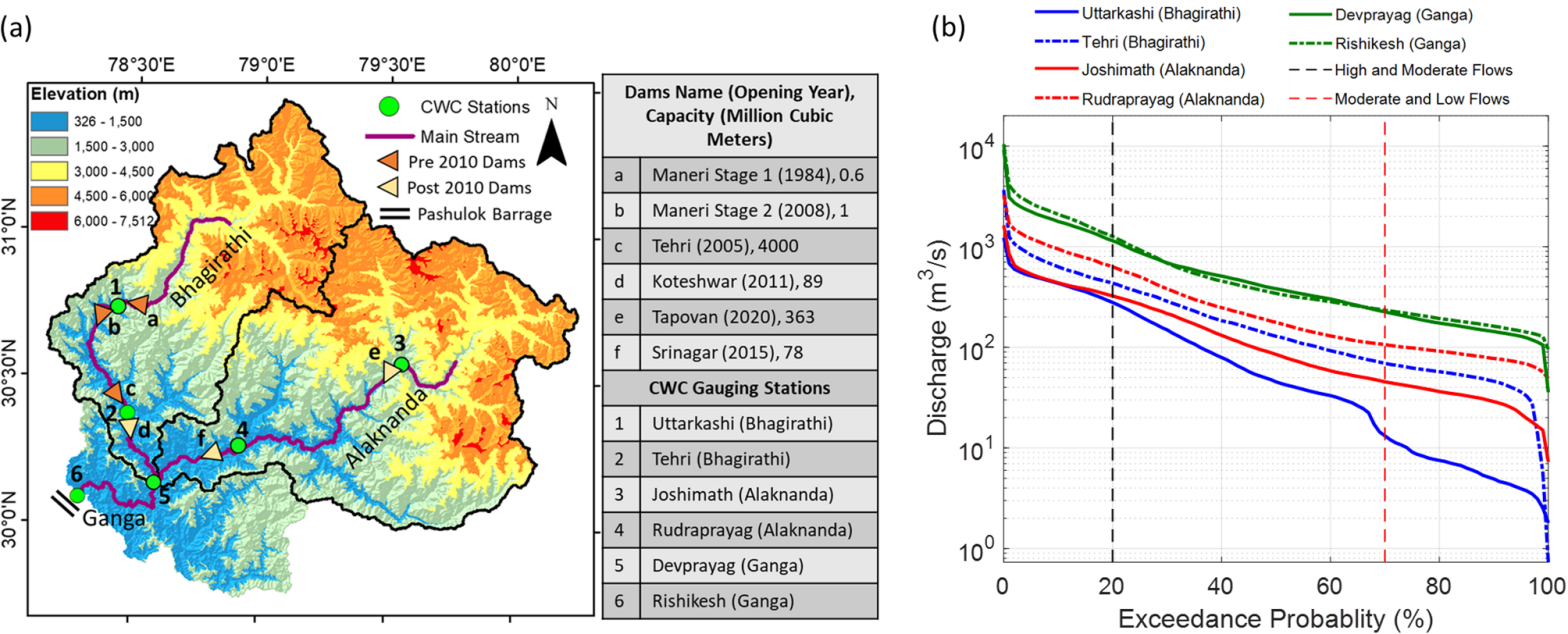
**(a)** Digital Elevation Model (DEM) of the UGB. The major stream network is also shown in the map using magenta lines. The CWC stations and dams are illustrated using filled green circles, orange and yellow triangles, respectively. Parallel black lines show the Pashulok barrage, located below the Rishikesh. Further, the name of dams and corresponding opening year is also shown in the side table. The QGIS Version 3.2 (https://qgis.org/en/site/forusers/download.html) was used to generate this figure. **(b)** Flow duration curves (FDCs) for the period 1970–2010. The solid and dashed blue, red and green FDC lines are shown for Bhagirathi (Uttarkashi and Tehri), Alaknanda (Joshimath and Rudraprayag) and downstream stations (Devprayag and Rishikesh), respectively.

**Figure 2:**
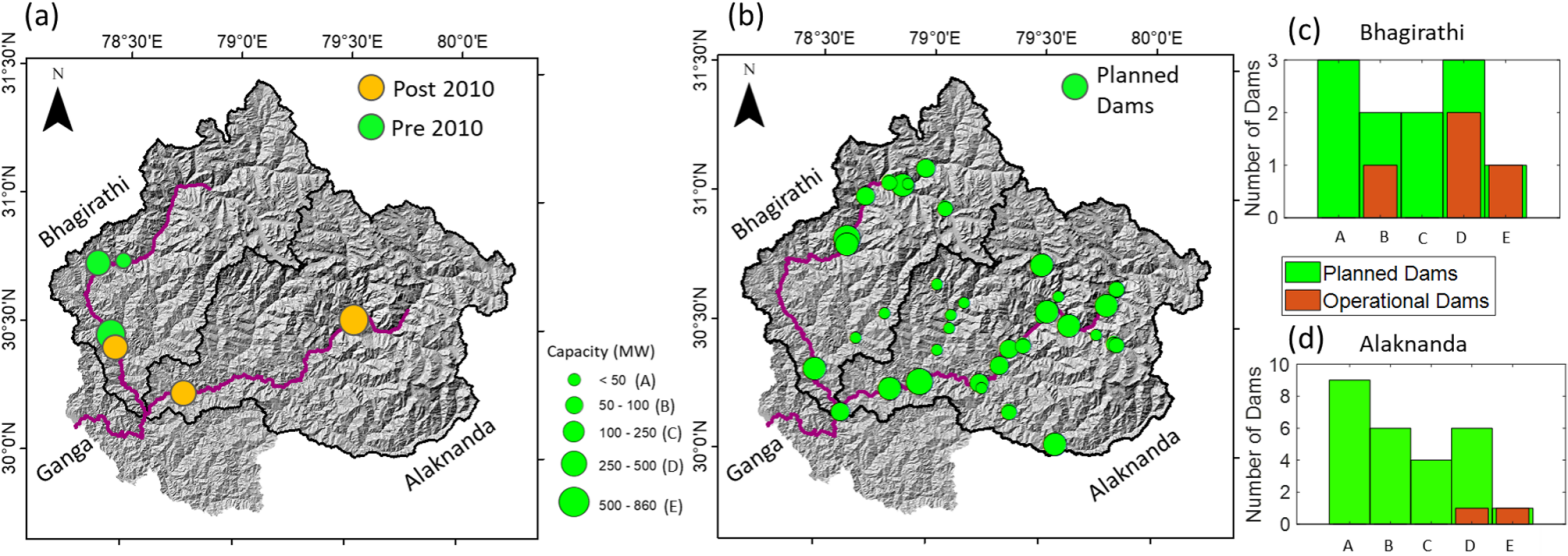
**(a)** Hydraulic structures in the UGB. The green and orange filled circles are showing the hydraulic structures commissioned pre-and post-2010 on the map. All these hydraulic structures’ circle sizes vary according to their hydropower generation capacity (MW). Further, A, B, C and D classes are defined based on each reservoir’s hydropower generation capacity. **(b)** The map shows planned hydraulic structures for the near future in the UGB. The QGIS Version 3.2 (https://qgis.org/en/site/forusers/download.html) was used to generate these figures. Furthermore, the bar plots show the current hydraulic structures and planned hydraulic structures for the near future in **(c)** the Bhagirathi and **(d)** the Alaknanda basin. The hill shade map of the UGB is used in the background.

The Ganga River has two major tributaries in the upper mountainous region. The western tributary, the Bhagirathi, originates from the Gangotri glacier (30.92° N, 79.08° E) at an elevation of about 4023 m. The eastern tributary, the Alaknanda, originates from the Satopanth glacier (30.79 N, 79.37 E), an elevation of about 4600 m. At Devprayag, both tributaries join to form the Ganga. Here, we have selected the Upper Ganga Basin (UGB) up to Rishikesh (area 21,000 km^2^) for this study (Fig. 1a). In the Bhagirathi basin, there are four dams, namely Maneri Stage 1, Maneri Stage 2, Tehri and Koteshwar dam (Figs. 1a, 2a), and most of these became operational before 2010. There are only two dams in the Alaknanda basin, namely Tapovan and Srinagar, which became operational after 2015 (Fig. 1a). Further, the Pashulok barrage is present downstream of Rishikesh. Furthermore, around 37 small and large dams are planned in the UGB (Fig. 2b), 11 in the Bhagirathi and 26 in the Alaknanda basin^44^ (Fig. 2c,d). The mean annual rainfall in the UGB ranges from 840 to 1990 mm (Fig. S1a). Almost 70% of the UGB receives 1000–1250 mm of rain annually, except for small patches in the western and eastern parts where more than 1500 mm of the mean annual rainfall occurs (Fig. S1a). Overall, the Indian Summer Monsoon (ISM) contribution across the UGB varies from 51 to 86% (Fig. S1b), leading to significant hydrological variabilities across the basin (Fig. 1b).

## Methodology

We used daily rainfall, discharge and suspended sediment load datasets for understanding the hydrological characteristics of the UGB. The details of the input dataset used in this study are listed in Table S1 (Supplementary). The exceedance probabilities were estimated in rainfall intensities, flows and sediment at each hydrological station. These estimates are depicted using the rainfall exceedance probability analysis, flow duration curve (FDC) and sediment duration curve (SDC). The daily rainfall, discharge, and suspended sediment datasets were divided into two periods—(1) 1971–1994 (pre-1995), and (2) 1995–2010 (post-1995). This temporal division was done based on the fact that the construction of a large hydraulic structure, i.e., Tehri dam (total capacity 4000 million cubic meters), started in 1995 in the Bhagirathi basin, and the frequency of flash flooding events increased in the UGB after 1995 (see Table S2). Hence, the anthropogenic and climate-induced alterations in the hydrology of the UGB could be captured by comparing the pre-1995 and post-1995 FDCs and SDCs. The FDCs and SDCs differences (in percentages) that showed for post-1995 were estimated with reference to pre-1995 FDCs and SDCs for all the gauge stations (see Figs. S3 and S4 in Supplementary). Further, the daily rainfall, discharge and sediments dataset for the 1971–2015 period at six stations are used for the hydrological analyses (Fig. 1a and Table S1). The first five years, i.e., 1971–1975, were selected in the Bhagirathi (at Uttarkashi and Tehri) and Alaknanda basin (at Joshimath and Rudraprayag) for initial reference conditions. However, the initial reference conditions at Devprayag and Rishikesh were considered to be 1976–1980 due to the unavailability of discharge and sediments data for the 1971–1975 period. In addition, 5-yearly rainfall magnitudes, FDCs and SDCs were also estimated and compared with each station’s reference condition to assess the temporal hydrological variations. The differences (in percentage) in 5-yearly rainfall magnitudes, FDCs and SDCs were calculated by subtracting selected 5-year periods with the initial reference condition and plotted on the 2D-contour plot for each station.

We also used the frequency analysis of extreme flows (annual maximum discharge) with the Generalized Extreme Value Type-1 (Gumbel) distribution^45^ at each station to estimate extreme discharge between 10- and 100-year return periods for pre-and post-1995. The Gumbel distribution for each station was selected based on Akaike Information Criterion (AIC) by comparing five widely used distributions, namely, (1) Lognormal, (2) Gamma, (3) Gumbel, (4) Weibull, and (5) Generalized Extreme Value (GEV; Table S3). The scale and location parameters of the Gumbel distribution were estimated using the Maximum Likelihood Estimation (MLE) method using the ‘FAmle’ package (https://github.com/tpetzoldt/FAmle) in R programming (see Fig. S5 in Supplementary). The differences between pre-and post-1995 extreme flows at different return periods were estimated and compared for all six gauging stations of the UGB (see Fig. S9 in Supplementary). It should be noted here that the credible extrapolation interval in flood frequency analysis is generally up to twice the record length. Hence, the 95% confidence bounds were also assessed and plotted in the return level graph for pre-1995, post-1995 and whole time series at each station of the UGB (see Figs. S6, S7 and S8 in Supplementary).

## Results and discussion

### Pre-and post-1995 hydrological scenarios

**Figure 3:**
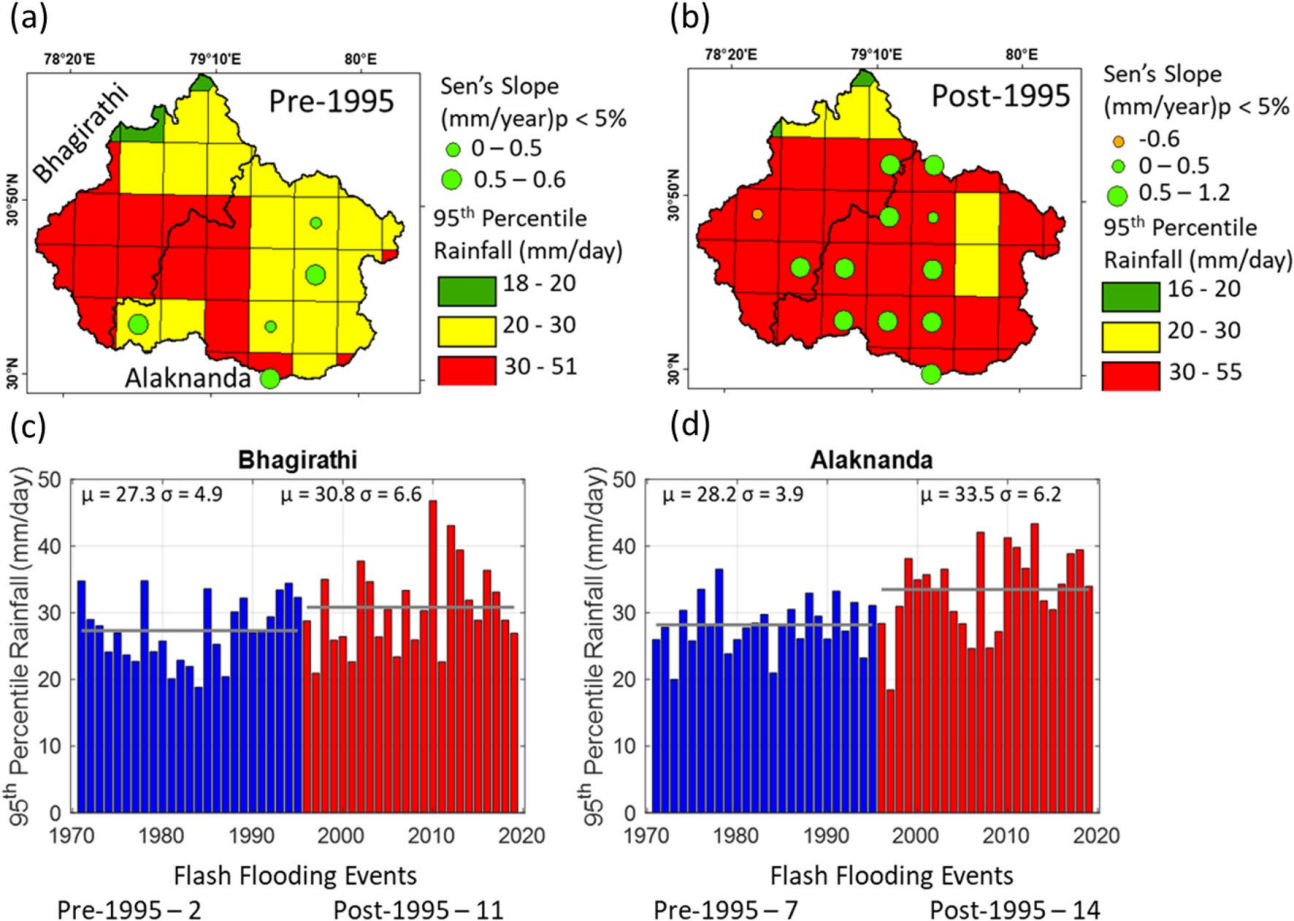
**(a)** Pre-and **(b)** post-1995 average 95th percentile rainfall magnitudes for 1970–2019. The different sizes of green filled circles represent the increasing Sen’s slope for the 95th percentile rainfall events at the 5% significance level. There is one orange-filled circle present in the Bhagirathi basin, which shows decreasing Sen’s slope for the 95th percentile rainfall events at a 5% significance level. **(c,d)** Shows bar plots 95th percentile rainfall of the Bhagirathi and Alaknanda basins for each year between 1970 and 2019. The blue and red bars show pre-and post-1995 annual cumulative rainfall magnitudes. The horizontal grey line shows the mean value of 95th percentile rainfall for the pre-and post-1995 period in both the bar plots. The mean (µ) and standard deviation (σ) of annual rainfall are also shown in both figures. Based on the available literature (see Table S2 in Supplementary Section), the extreme flooding events are also mentioned for the corresponding plots of the Bhagirathi and Alaknanda River basins.

The UGB has experienced a widespread increase in high-intensity rainfall events after 1995 (Fig. 3a,b). These are statistically increasing (p < 0.05) trends predominantly in the Alaknanda basin (Fig. 3b). It is also noted that the Alaknanda basin has been experiencing a rising trend of high-intensity rainfall events compared to the Bhagirathi basin since 1995. The observed records also suggest an increase in extreme flooding events in the UGB (Fig. 3c,d and Table S2). A total of 9 and 25 extreme flooding events are reported for the two basins together during the pre-and post-1995 period, respectively. The Bhagirathi basin has experienced 2 and 11 extreme flooding events during the pre-and post-1995 period (Fig. 3c and Table S2). The Alaknanda basin has undergone 7 and 14 extreme flooding events during the pre-and post-1995 period (Fig. 3d and Table S2). In terms of temperature, the Bhagirathi and the Alaknanda basins show statistically significant increasing trends. However, these increasing trends detected by the statistical tests are likely driven by the step-change that occurred between pre-and post-2000, possibly suggesting a shift in the instrumentation (Fig. S2a,c). Further, there is no step-change or significant trend detected in the maximum temperature for both the basins (Figs. S2b,d).

**Figure 4:**
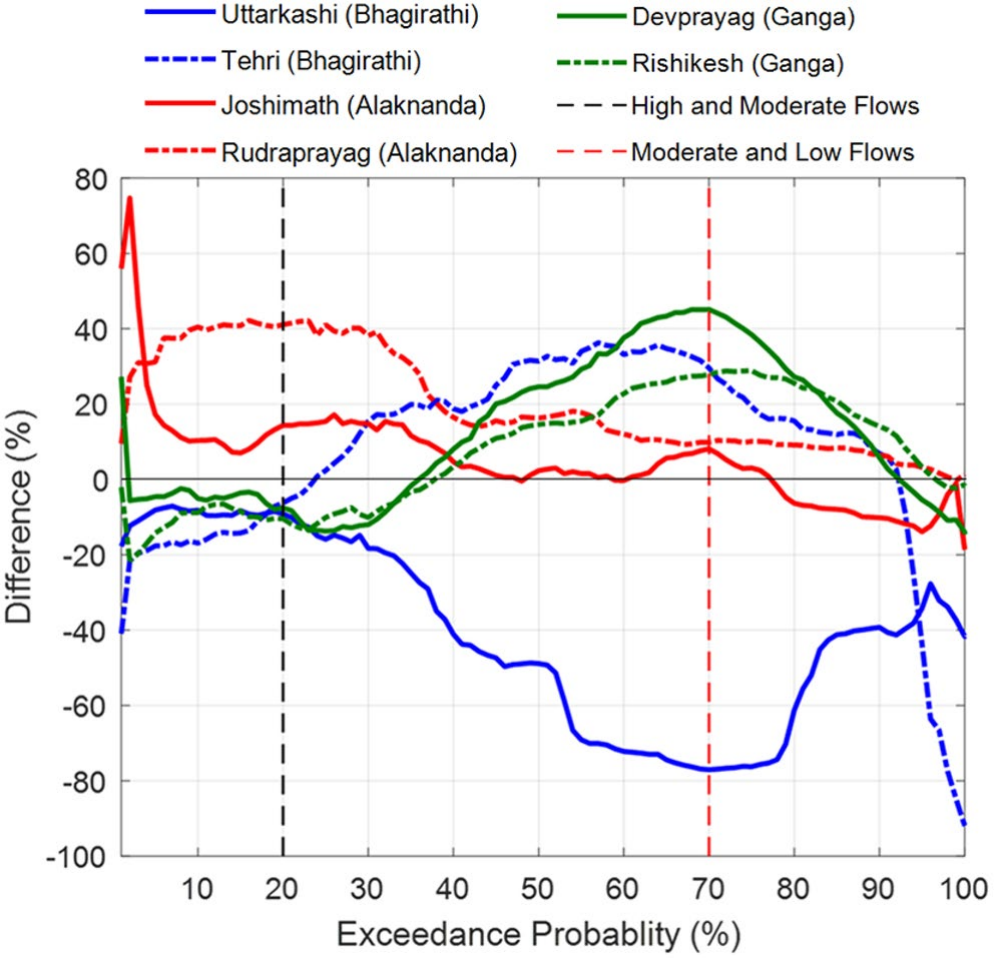
Difference between post-and pre-1995 flow duration curves (FDCs). These differences (%) are plotted for Uttarkashi (blue line), Tehri (dashed blue line), Joshimath (red line), Rudraprayag (dashed red line), Devprayag (green line) and Rishikesh (green dashed line) stations of the UGB. The division between high and moderate (at 20%) and moderate and low (at 70%) flows are shown by dashed black and dashed red vertical lines.

**Figure 5:**
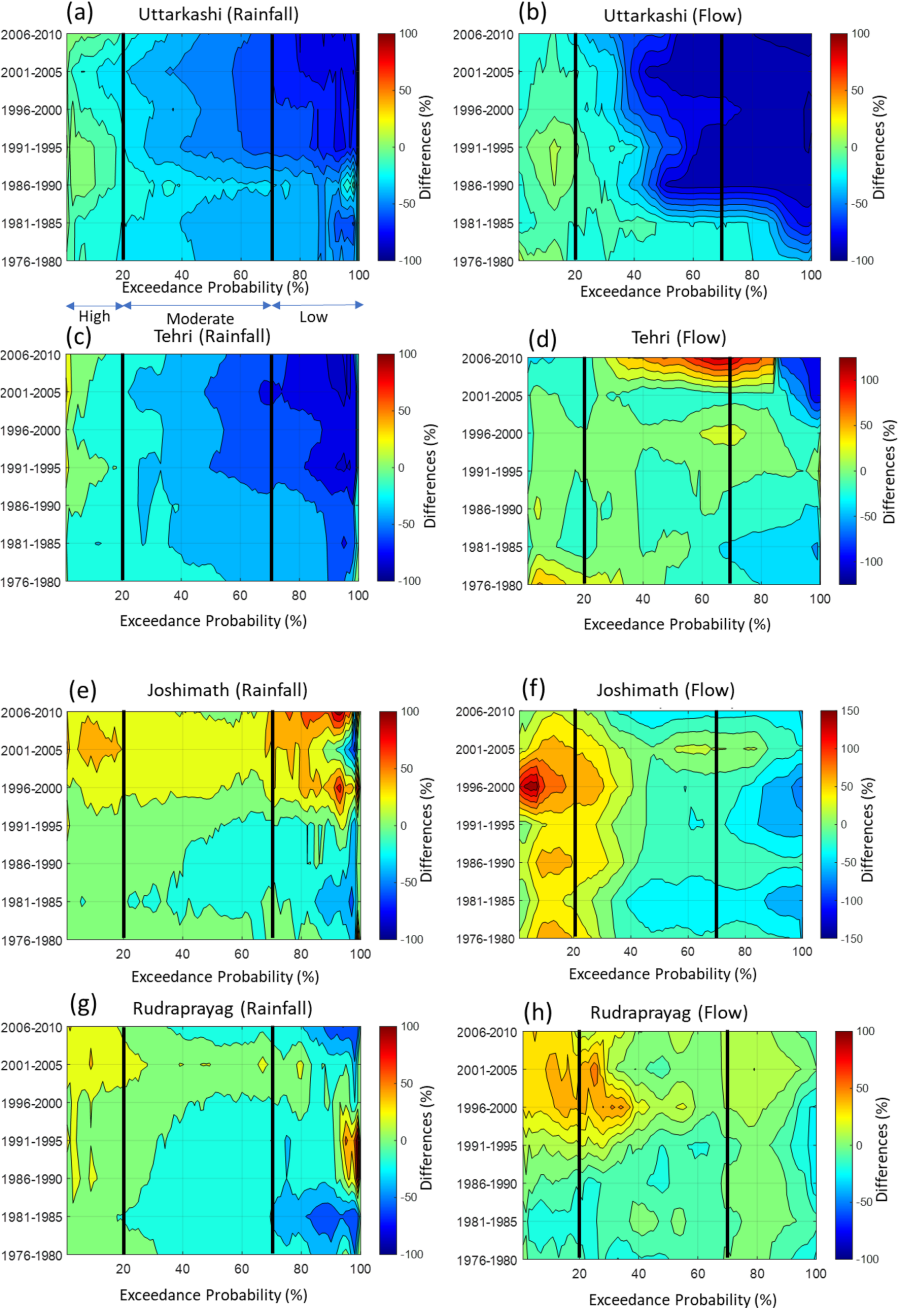

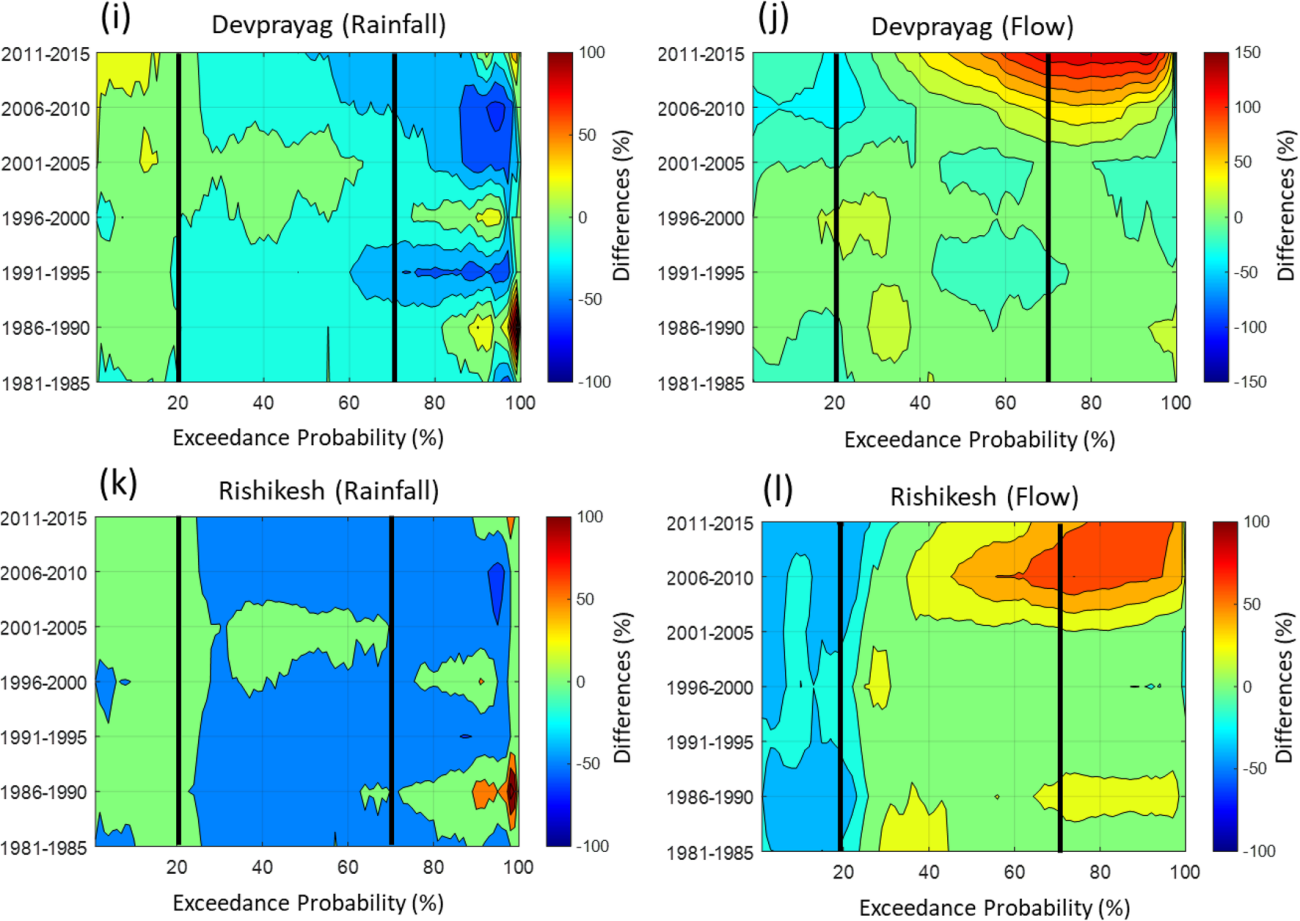
5-yearly differences in rainfall and flow duration curves (FDCs) are plotted using 2d contour plot for each station. The high (20% <), moderate (20–70%) and low (> 70%) flows are divided by black vertical lines. **(a)** The Uttarkashi station shows 90% reduction in low rainfall. The high rainfall slightly increased (up to 10% since 1986). **(b)** The Uttarkashi station shows up to 90% reduction (1981–1985) in low flows. The high flows also decreased up to 30%. **(c)** The Tehri station shows a reduction of 100% in low rainfall throughout the period. There is no anomalous behaviour observed in low and moderate rainfall magnitudes after 2005 at Tehri. **(d)** The Tehri station shows a reduction of up to 50% in low flows until 1990. After 2000, the reduction in low flows up to 85% is also appeared here. The high flows increased by 50% after 2005. The moderate flows have been increased up to 80% after 2005. **(e)** The Joshimath station shows increasing high, low and moderate rainfall magnitudes after 1996. The high and low magnitude rainfalls are increased up to 30% and 50%, respectively. **(f)** The Joshimath station shows a reduction from 20 to 70% in low and moderate flows. The high flows increased by 20% from the reference condition. **(g)** The Rudraprayag station shows increasing high magnitude rainfall by 30% from 1996. The moderate rainfall magnitudes have also slightly increased post-1995. **(h)** The Rudraprayag station shows a 10–20% reduction in all flows until 1995. The high flows have been increased 20–40% after 1995. The high rainfall magnitudes have increased up to 10% at **(i)** Devprayag and **(k)** Rishikesh stations. However, the high rainfall magnitudes have increased steeply (up to 30%) after 2005 at Devprayag than Rishikesh. The low and moderate rainfall magnitudes have decreased from the reference period at both stations. However, the percentage reductions in low and moderate rainfall magnitudes are slightly higher for the Rishikesh (up to 50%) than Devprayag (20% to 30%). The major changes in low and moderate flows up to 80% and 40% appeared at **(j)** Devprayag and **(l)** Rishikesh after 2005.

In the Bhagirathi basin, the difference of post-and pre-1995 FDCs suggests a substantial reduction of up to 80% in very low flows (> 90% exceedance probability) at Uttarkashi (Fig. 4 and Table S4). The 5-yearly rainfall magnitude differences suggest around 50–100% reduction in low and moderate magnitude rainfall events from the reference period (Fig. 5a). Further, the 5-yearly differences of FDCs reveal around 60–90% decline in low and moderate flows at Uttarkashi (Fig. 5b). Coincidently, upstream of Uttarkashi, the Maneri Stage 1 dam construction was started in the 1960s, and this dam became operational in 1984 (Fig. 1a). Therefore, a very sharp reduction in the low and moderate flows from the reference condition can be directly correlated to the operation of the Maneri Stage 1 dam. However, a decrease in the magnitude of low and moderate rainfall after 1991 (Fig. 5a) further attenuated the low and moderate flows at Uttarkashi station (Fig. 5b). Furthermore, the Maneri Stage 2 dam, located immediately downstream of the Uttarkashi, became operational in 2008 (Fig. 1a), and this might have also started influencing the hydrology at Uttarkashi since then.

In contrast, at Tehri, the flows between 30 and 85% exceedance probability (moderate to low flows) have increased by 80% in post-1995 (Figs. 4, 5d) with reference to pre-1995. The difference in the FDCs of pre-and post-1995 FDCs suggests that the moderate and low flows increased rapidly downstream of the Tehri dam after becoming operational (Fig. 4). Additionally, the very low flows (> 90% exceedance probability) have decreased by 90% at Tehri (Fig. 5d). The 5-yearly differences in rainfall magnitudes suggest that the moderate and low rainfall magnitudes decreased significantly after 1991 (30% to 100%; Fig. 5c). The magnitude of very high rainfall (< 10% exceedance probability) has increased up to 30% at Tehri. In comparison, the characteristics of high and moderate flows behaviour after 1995 do not match those of high and moderate rainfall magnitudes (Fig. 5c,d). Such anomalous hydrological behaviour of the Bhagirathi River at Tehri strongly suggests alteration of flow regime caused by the Tehri dam operation. Hence, the existence of dams in the Bhagirathi basin has reduced the extreme flows and floods downstream. Further, the moderate and low flows have significantly increased up to 125% post-2005. These abrupt increments and decrements in the flows are not observed anywhere in the UGB (Figs. 4, 5). Besides, the upstream (Uttarkashi) and downstream (Tehri) stations in the Bhagirathi behave differently. These distinct and abrupt hydrological behaviour indicate the significant impact of Maneri and Tehri dams in modifying the water outflux from the Bhagirathi basin.

In the Alaknanda basin, there was no dam before 2010 (Fig. 1a). The Srinagar dam and Tapovan dam became operational in 2015 and 2020, respectively^46^. Thus, the possibility of anthropogenically altered river flow due to reservoir operation can be ruled out before 2010. The differences in the post-and pre-1995 FDCs suggest that the high and moderate flows increased at Joshimath and Rudraprayag (Fig. 4 and Table S4). These differences are more predominant at Rudraprayag (up to 40%). In particular, the 5-yearly differences of FDCs from their reference condition also reveal that the high flows increased significantly after 1995 at both locations (Fig. 5f,h). High flows at Rudraprayag show an increasing trend until 2010. However, a sudden increase (up to 100% or doubled) in high flows is observed between 1995 and 2005 for Joshimath station. The 5-yearly rainfall differences suggest increasing high magnitude rainfall after 1995 at Joshimath (up to 150%) and Rudraprayag (up to 50%; Fig. 5e,g). It is also evident that the increasing trends (p < 0.05) of high rainfall intensities (95th percentile) have doubled (0.6 mm/y in pre-1995 and 1.2 mm/y in post-1995) and are more widespread in the Alaknanda basin (Fig. 3b). Therefore, we argue that the increase in the high flows is linked to increasing intensities of high-intensity rainfall events in the Alaknanda basin. Further, the reported extreme events strongly suggest an increase of extreme rainfall linked to flooding events in this basin (Fig. 3d), which have doubled (7 events in pre-1995 and 14 events in post-1995). These observations indicate that the changing climatic conditions, remarkably increasing trends of high-intensity rainfall events primarily controlled the hydrology of the Alaknanda basin until 2010. However, after the opening of the Srinagar dam (in 2015) and the Tapovan dam (in 2020; Fig. 1a), the current flows might have been anthropogenically modified in addition to the impact of changing climatic conditions.

In the downstream reaches, high and very low flows (20% < and > 90% exceedance probability) are governed by increasing and decreasing flows from the Alaknanda and Bhagirathi basins, respectively (Fig. 4). However, the moderate and low flows (20–90% exceedance probability) at Devprayag and Rishikesh are predominately influenced by the moderate flows coming out from the Tehri (Bhagirathi). The 5-yearly FDCs differences at Devprayag and Rishikesh further suggest a substantial increase in moderate and low flows (> 40%), particularly after 2005 (Fig. 5j,l and Table S4). However, there are no such substantial increments observed in the moderate and low rainfall magnitudes at both downstream stations (Fig. 5i,k). These patterns strongly correlate with Tehri’s post-2005 moderate and low flows fluctuations (Fig. 5d). Therefore, these observations suggest that the Tehri dam's water flux increased the moderate and low flows at Devprayag and Rishikesh since 2005, although these fluctuations became more significant post-2010 (Fig. 5j,l).

**Figure 6:**
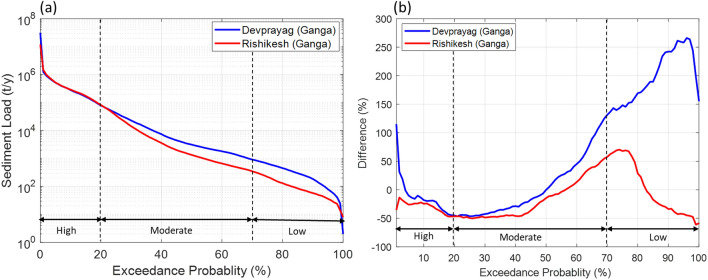
**(a)** Sediment duration curves (SDCs) of Devprayag (blue) and Rishikesh (red) for the period 1970–2015. The high sediment fluxes (20% <) are comparable for both stations. However, the peak sediment fluxes are slightly higher for the Devprayag station. Further, the moderate (20–70%) and low sediment fluxes (> 70%) are an order of magnitude higher for Devprayag than the Rishikesh station. **(b)** Difference (%) between pre-and post-1995 SDCs of Devprayag (blue) and Rishikesh (red) stations. The peak sediment flows have been increased (120%) at Devprayag. In contrast, the peak sediment fluxes have been decreased (− 25%) at Rishikesh. Furthermore, the moderate sediment fluxes have also been reduced (up to 50%) at both stations. However, moderate and low sediment fluxes greater than 50% and 55% exceedance probability are increased from their pre-1995 values at both stations.

Further, sediment duration curves (SDCs) suggest that high sediment fluxes are nearly similar for downstream stations. However, moderate and low sediment fluxes are an order of magnitude higher for the Devprayag and Rishikesh stations (Fig. 6a). These differences indicate that a significant amount of sediments has been deposited between Devprayag and Rishikesh, possibly due to reduced inflow. The post- and pre-1995 differences suggest that high sediment fluxes (50% < exceedance probability) have decreased up to 50% at both locations (Fig. 6b). These differences indicate that a considerable part of high-magnitude sediment flux is deposited upstream of Devprayag (possibly in the Tehri and Maneri reservoirs; Fig. 1a) and not reaching the main channel downstream. Moderate and low sediment fluxes (> 50% exceedance probability) have increased tremendously at Devprayag (up to 260%) and Rishikesh (up to 70%; Fig. 6b). These incredibly increasing amounts can be linked to sediment reworking caused by abrupt behaviour of moderate and low flows at Devprayag and Rishikesh governed by the reservoir-induced increase of moderate and low flows (Figs. 4, 5d,j,l). Therefore, these observations strongly suggest that the Tehri dam in the Bhagirathi basin plays a crucial role in determining the hydrological variability of the downstream UGB region.

### Role of natural and anthropogenic stressors on changing extreme flows

**Figure 7:**
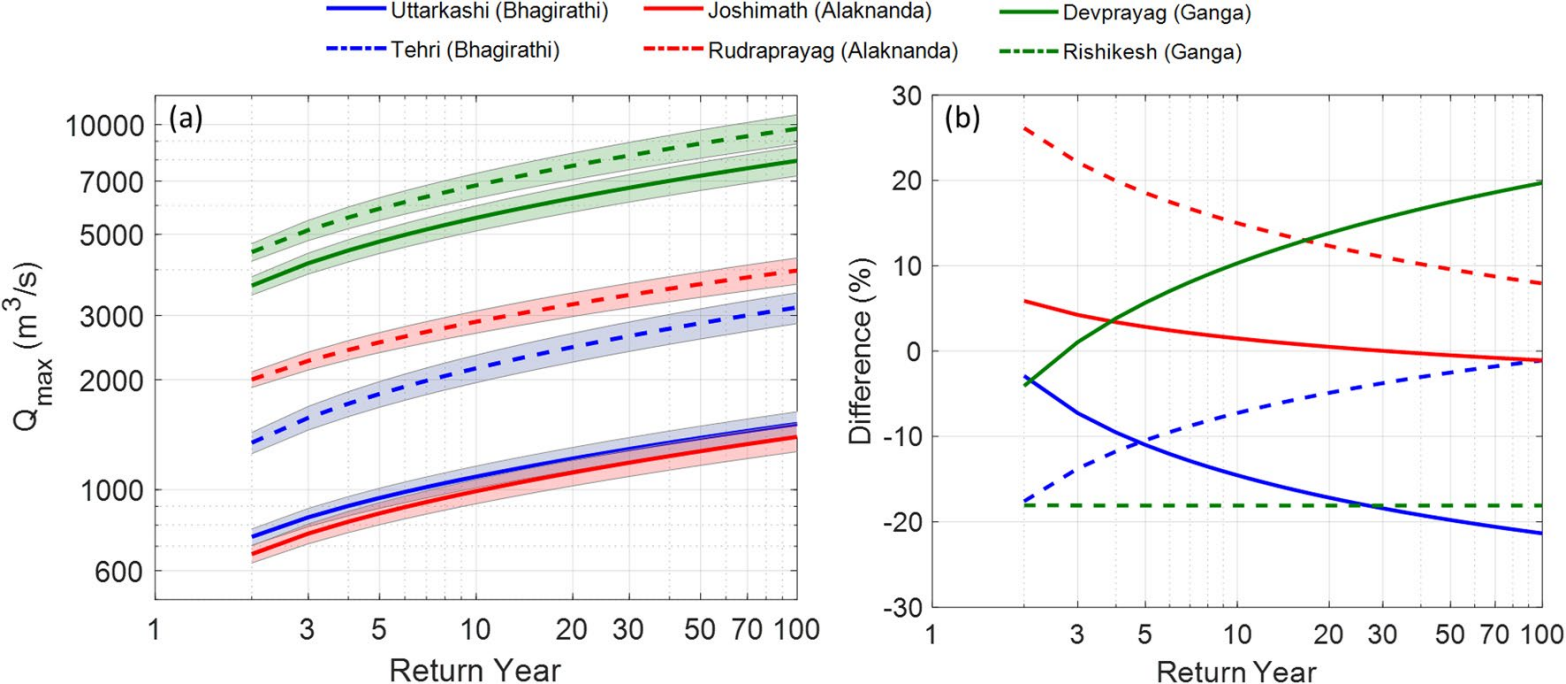
**(a)** Extreme flows at different return periods at the six stations of the UGB. The Rishikesh (downstream station) and Joshimath (upstream Alaknanda basin) stations show the highest and lowest extreme flows at different return periods. The standard errors of the scale and location parameters of the Gumbel distribution are used to predict the error bound and shown using shaded regions around each return level curve. The details of 95% confidence bounds around the prediction of return level for each station are given in Supplementary. **(b)** Post-and pre-1995 differences of extreme flows at different return periods for different stations.

Frequency analysis of extreme flooding events suggests that the UGB has experienced contrasting responses due to natural and anthropogenic forcing. For instance, at Uttarkashi and Tehri, the Bhagirathi basin exhibits a total reduction of extreme flows at different return periods. Around -14.5%, -17.9% and -21.3% reductions are observed in the magnitude of 10, 50 and 100-year return period floods at Uttarkashi (Fig. 7a,b and Table S4). In comparison, around -7.3%, -2.5% and -1.1% reductions are observed in the magnitude of 10, 50 and 100-year return period floods at Tehri (Fig. 7b). Such decreasing extreme flows in the Bhagirathi basin are primarily governed by two major factors: (1) presence of small and large hydraulic structures such as Maneri Stage 1, Maneri Stage 2, Tehri and Koteshwar dam (Fig. 1a), and (2) no significantly increasing or decreasing trends in the high-intensity rainfall events (Fig. 3a,b).

In contrast, the Alaknanda river at Joshimath and Rudraprayag show an increase of extreme flows at different return periods. For instance, around 1.5%, -0.5% and -1.1% differences are observed in the magnitude of 10-, 50-and 100-year return period floods at Joshimath (Fig. 7b and Table S4). In comparison, around 15%, 9.6% and 7.9% increments are observed in the magnitude of 10-, 50-and 100-year return period floods at Rudraprayag (Fig. 7b and Table S4). Therefore, the extreme flows and flooding events in the Alaknanda basin (particularly at Rudraprayag) are primarily governed by two major factors: (1) no hydraulic structures present before 2010 (Fig. 1a), and (2) widespread increasing high-intensity rainfall in this basin (Fig. 3a,b,d). Further, the oldest dam, Maneri Stage 1, has been operational since 1984 in the Bhagirathi basin, whereas the Srinagar dam and Tapovan dam in the Alaknanda became operational in 2015 and 2020, respectively. Therefore, we argue that the increasing number of hydraulic structures after 2015 has also impacted the extreme flows of the Alaknanda basin.

The downstream stations of the UGB behave differently when we compare the pre-and post-1995 extreme flows at different return periods. For instance, we document an increment of 10.3%, 17.5% and 19.7% in the magnitude of 10-, 50- and 100-year floods at Devprayag in the post-1995 period (Fig. 7b and Table S4). However, the Rishikesh station records an -18.1% reduction in the magnitude of 10-, 50- and 100-year floods in the post-1995 period (Fig. 7b and Table S4). A reduction in extreme flow magnitudes is possibly because of flow reduction caused by the Pashulok barrage downstream of the Rishikesh station. We have also observed a significant reduction in high magnitude stream flows at Rishikesh than Devprayag station (Fig. 5j,l). The post-1995 extreme flows have decreased in the Bhagirathi basin but increased in the Alaknanda (Fig. 7b). Therefore, a rise in extreme flooding events at Devprayag station is primarily governed by the changes in hydrometeorological conditions in the Alaknanda basin. The widespread increase in high-intensity rainfall in the Alaknanda basin and the reservoir-induced flow alterations are the primary drivers of these changes in observed extreme flow at Devprayag and Rishikesh (Fig. 3a–c).

It is also observed that the downstream (Rudraprayag) region of the Alaknanda shows an incremental difference of up to 15% in the extreme flows (Fig. 7b) which makes the entire downstream Alaknanda basin vulnerable to extreme flooding events in the near future. One such event was reported recently (in February 2021) near Joshimath, which destroyed the Tapovan dam^47^. Downstream of Rishikesh, the Ganga River debouches into the alluvial plains (Fig. 1a), where several populous cities are situated. Therefore, these are the vulnerable regions where around 20% increase in extreme flooding events (at Devprayag) might enhance the flood risk manifold. Further, the Pashulok barrage downstream of Rishikesh was constructed in 1980 based on the past extreme flow information until then. However, the changing climatic conditions in the Alaknanda basin, and hence, an increase of 10–20% in extreme flows, might severely affect the operations of such structures.

### Increasing anthropogenic activities and their future impacts

Overall, these hydrological analyses indicate that the flow in the Bhagirathi basin has been anthropogenically modified owing to the presence of several large and small dams (Figs. 1a, 4, 5b,d). In particular, low and moderate flows, which occur primarily during pre- (Jan-May) and post-monsoon (Oct-Dec) periods, are significantly impacted (Figs. 4, 5b,d). The Alaknanda was a free-flowing river before the Srinagar dam was commissioned in 2015 (Figs. 1a, 4, 5f,h), followed by the Tapovan dam in 2020 (Fig. 1a,b). However, our data records could not capture these hydrological alterations at Joshimath and Rudraprayag in the Alaknanda (see Table S1). Recent hydrological records can be further used to verify these hydrological changes. We have demonstrated that the present low and moderate flows coming out from Devprayag and Rishikesh (downstream of the UGB) are entirely modified anthropogenically (Figs. 4, 5j,l). The interventions have severely affected the upstream and downstream hydrology and geomorphology of the Bhagirathi basin (Figs. 5j,l, 6a,b).

Around 11 and 26 additional dams of different power generation capacities in the Bhagirathi and Alaknanda basin, respectively, are planned^44^ (Fig. 2b–d). These planned hydraulic structures will be located on several small and large tributaries of the UGB (Fig. 2b). These structures are likely to impact the low and moderate flows of the UGB further, as demonstrated in the case of the Bhagirathi basin. Additionally, the increasing number of dams will also influence the sediment transport processes across the UGB (Fig. 6b). Further, a significant increase in the high magnitude flows is also observed in the Alaknanda River basin and Devprayag (Fig. 7a,b). The impact of changing climatic conditions are more predominant in the Alaknanda basin (Fig. 3a,b). Our extreme frequency analysis also suggests an increase in the magnitude of extreme flows for different return periods in the Alaknanda basin (Fig. 7a,b). Further, the observed records indicate an increase in the frequency of extreme flood events in the UGB, especially in the Alaknanda basin (Fig. 3a,b). During the flash flood event at Joshimath in February 2021^47^, high discharges were quickly managed because of the lean condition of the mainstream. However, if this event had occurred during the monsoon season, it could pose a severe flood risk in the downstream regions. In the past, the UGB region also witnessed the June 2013 Kedarnath disaster when rainfall magnitudes crossed a 111-year return period and produced a massive flood in the monsoon period^26^ (Table S2). Thus, the changing extremity of streamflow in the UGB poses serious impacts on the hydraulic structures that need critical assessment and design modifications.

## Conclusions

The Ganga River is the lifeline for close to half a billion people in the northern Indian region. During the twentieth century, the hydrology of the basin has been significantly modified owing to increasing anthropogenic interventions and changing climatic conditions. In particular, the upper Ganga basin (UGB) has witnessed modifications in the flow regime owing to several small and large hydraulic structures, particularly in the Bhagirathi basin (western tributary). In contrast, the Alaknanda basin (eastern tributary) has experienced increasing magnitudes of extreme rainfall events from 1970 to 2019. Therefore, the flow modifications in these basins have been influenced by different factors. Our results suggest that the reduction in rainfall magnitudes, Maneri dam in upstream and Tehri dam in downstream exert primary controls on the flows in the Bhagirathi. As a result, low and moderate flows increased at Tehri by 125%. In addition, the post-1995 extreme flows at different return periods have decreased by -21.3% in the Bhagirathi basin. Further, the Alaknanda basin was a free-flowing river until 2015. The extreme flows at different return periods have increased by 8–15% in the Alaknanda basin, primarily because of increasing high-intensity rainfall events post-1995. Therefore, the Alaknanda basin has witnessed some extreme flash flood events in recent years. Simultaneously, the downstream reaches experience anthropogenically modified low and moderate flows that are attributed to Tehri and other dams during pre-and post-monsoon months.

Our results further indicate that a significant amount of sediments transported during high flows are trapped in the Tehri and Maneri reservoirs in the Bhagirathi basin. Therefore, hydraulic structures have significantly disrupted the upstream–downstream geomorphologic linkages, thereby impacting the channel morphology in the downstream reaches as observed in several regions^48–51^. Furthermore, several hydraulic structures such as the Pashulok barrage were designed based on analysis of past extreme floods. However, the increasing magnitude of extreme flows (10-20%), particularly at Devprayag, might also affect the functioning of the Pashulok barrage during peak monsoon periods. The downstream regions also experience reservoir-induced flow increments during pre-and post-monsoonal months. Overall, the results obtained from this work should help in sustainable river basin management and encourage more serious work toward a better understanding of hydrology, ecology, and geomorphology in the UGB.

## Supplementary Information

Supplementary Information.
