# Supplementary material for: Modified hydrologic regime of upper Ganga basin induced by natural and anthropogenic stressors
Source: Sci Rep. 2021 Sep 30;11:19491. doi: 10.1038/s41598-021-98827-7 (PMC8484260; doi:10.1038/s41598-021-98827-7)
Supplement: Supplementary file 1 — Supplementary Information. [file 41598_2021_98827_MOESM1_ESM.docx]

Modified Hydrologic Regime of Upper Ganga Basin Induced by Natural and Anthropogenic Stressors

Somil Swarnkar^1^, Pradeep Mujumdar^1^ and Rajiv Sinha^2^

1. Interdisciplinary Centre for Water and Research (ICWaR), Indian Institute of Science (IISc), Bangalore, India
2. Department of Earth Sciences, Indian Institute of Technology Kanpur (IITK), Kanpur, India

Corresponding author – Prof. Pradeep Mujumdar ([pradeep@iisc.ac.in](file:///F:\CRR\Misc%20work%20of%20PPM\Web%20page\pradeep@civil.iisc.ernet.in))

**Supplementary**

**Table S1** Input dataset used in this study

| S. No. | Input Data | Agency | Spatial and Temporal Resolution | Time Period | Reference |
| --- | --- | --- | --- | --- | --- |
| 1 | Digital Elevation Model (DEM) | Shutter Radar Topography Mission (SRTM) | 90 meters | 2001 | Jarvis et al. (2008) |
| 2 | Rainfall | Indian Meteorological Department (IMD) | 0.25^o^ (Daily) | 1970-2019 | Pai et al. (2014) |
| 3 | Minimum and Maximum Temperature |  | 1^o^ (Daily) | 1970-2019 | Srivastava et al.  (2009) |
| 4 | Discharge | Central Water Commission (CWC) | Daily | 1971-2015 |  |
| 5 | Suspended Sediment Load |  |  |  |  |

**Table S2** Extreme Flash Flooding Events occurred in the Alaknanda and Bhagirathi basin

| S. No. | Alaknanda Basin | | | S. No. | Bhagirathi Basin | | |
| --- | --- | --- | --- | --- | --- | --- | --- |
|  | Date | Region | Referenc |  | Date | Region | Reference |
| 1 | 20-Jul-1970 | Chamoli | Joshi and Kumar (2006) | 1 | 4-5 Aug-1978 | Uttarkashi | Prakash (2015) |
| 2 | 17-Jun-1979 | Chamoli |  | 2 | 24-25 June-1980 | Uttarkashi |  |
| 3 | 17-Aug-1979 | Rudraprayag |  | 3 | Jul-2000 | Uttarkashi |  |
| 4 | 31-Jul-1982 | Manda Khal |  | 4 | 31-Aug-2001 | Tehri | Naithani et al. (2002b) |
| 5 | 9-Jul-1990 | Neelkhand |  | 5 | 10-Aug-2002 | Tehri | Sah et al. (2003) |
| 6 | 16-Aug-1991 | Chamoli | Joshi and Maikhuri (1997) | 6 | 19-20 Aug-2002 | Uttarkashi | Khanduri (2020) |
| 7 | Jul-1993 | Chamoli | Sati (2007) | 7 | 29-Aug-2003 | Uttarkashi |  |
| 8 | 13-Aug-1995 | Bhintai | Joshi and Kumar (2006) | 8 | 27-Aug-2005 | Uttarkashi |  |
| 9 | 11-19 Aug-1998 | Rudraprayag | Naithani (2001) | 9 | 17-19 Sep 2010 | Uttarkashi | Sharma (2012) |
| 10 | 16-Jul-2001 | Rudraprayag | Naithani et al. (2002a) | 10 | 3-Aug-2012 | Uttarkashi | Gupta et al. (2013) |
| 11 | 24-Sep-2004 | Badrinath | NIDM (2015) | 11 | 16-17 June 2013 | Uttarkashi | NIDM (2014) |
| 12 | 29-30 Jun-2005 | Chamoli | Asthana and Asthana (2014) | 12 | 28-May-2016 | Tehri | Dimri et al. (2017) |
| 13 | 21-Jul-2005 | Rudraprayag | Khanduri (2020) | 13 | 18-Aug-2019 | Uttarkashi | Khanduri and Sajwan (2019) |
| 14 | 11-Aug-2006 | Chamoli |  | 14 | 10-Aug-2020 | Uttarkashi |  |
| 15 | 26-Jul-2006 | Rudraprayag |  |  | | | |
| 16 | 17-19 Sep 2010 | Rudraprayag | Sharma (2012) |  |  |  |  |
| 17 | 13-Sep-2012 | Rudraprayag | DMMC (2012) |  |  |  |  |
| 18 | 16-17 June-2013 | Kedarnath, Badrinath, Guptkashi | NIDM (2014) |  |  |  |  |
| 19 | 19-Jul-2018 | Joshimath | Khanduri (2020) |  |  |  |  |
| 20 | 8-9 Aug-2019 | Chamoli |  |  |  |  |  |
| 21 | 6-7 Sep-2019 | Joshimath |  |  |  |  |  |

**Table S3** AIC values for different distributions fitted in pre-and post-1995 peak discharge time series at each station of the UGB

| **Distribution** | **Uttarkashi** | | **Tehri** | | **Joshimath** | | **Rudraprayag** | | **Devprayag** | | **Rishikesh** | |
| --- | --- | --- | --- | --- | --- | --- | --- | --- | --- | --- | --- | --- |
|  | pre-1995 | post-1995 | pre-1995 | post-1995 | pre-1995 | post-1995 | pre-1995 | post-1995 | pre-1995 | post-1995 | pre-1995 | post-1995 |
| **Lognormal** | 342.2 | 195.7 | 380.6 | 232.1 | 337.3 | 207.0 | 382.0 | 228.2 | 428.3 | 384.5 | 444.8 | 386.1 |
| **Gamma** | 339.4 | 195.8 | 382.9 | 231.3 | 336.0 | 209.2 | 382.0 | 228.2 | 428.9 | 386.3 | 445.6 | 387.3 |
| **Gumbel** | 338.7 | 196.0 | 380.0 | 230.9 | 334.2 | 205.9 | 382.1 | 228.3 | 428.3 | 384.7 | 444.7 | 385.3 |
| **Weibull** | 337.8 | 196.7 | 390.5 | 230.9 | 335.3 | 214.4 | 385.1 | 229.5 | 432.5 | 390.1 | 450.4 | 396.9 |
| **GEV** | 339.4 | 197.6 | 381.4 | 233.1 | 336.3 | 206.4 | 383.7 | 238.9 | 430.3 | 386.2 | 447.5 | 389.9 |

**Table S4** Pre-and Post-1995 differences in flow duration curves, sediment duration curves and extreme frequency analysis

| S. No. | Station Name | Basin Name | Differences in Flow Duration Curves | | | Differences in Sediment Duration Curves | | | Differences in Extreme Flows | | |
| --- | --- | --- | --- | --- | --- | --- | --- | --- | --- | --- | --- |
|  |  |  | High | Moderate | Low | High flows | Moderate flows | Low flows | 10-Year | 50-Year | 100-Year |
| 1 | Uttarkashi | Bhagirathi | -17% to -10% | -75% to -12% | -75% to -28% | - | - | - | -14.5% | -17.9% | -21.3% |
| 2 | Tehri |  | -40% to -5% | -5% to 38% | -100% to 38% | - | - | - | -7.3% | -2.5% | -1.1% |
| 3 | Joshimath | Alaknanda | 15% to 75% | 0% to 18% | -40% to 8% | - | - | - | 1.5% | -0.5% | -1.1% |
| 4 | Rudraprayag |  | 10% to 40% | 10% to 40% | 0% to 10% | - | - | - | 15% | 9.6% | 7.9% |
| 5 | Devprayag | Ganga | -8% to 28% | -10% to 40% | -60% to 40% | -50% to 125% | -50% to 125% | 125% to 300% | 10.3% | 17.5% | 19.7% |
| 6 | Rishikesh |  | -20% to -3% | -13% to 28% | -30% to 28% | -45% to -15% | -50% to 60% | -60% to 70% | -18.1% | -18.1% | -18.1% |


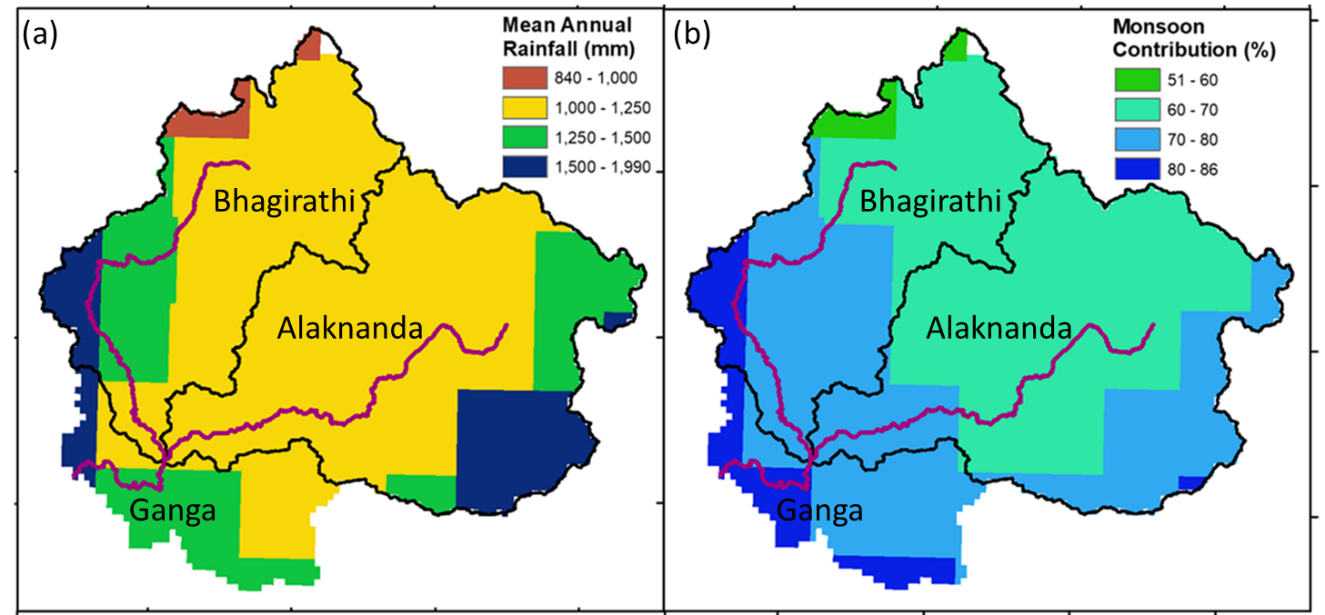


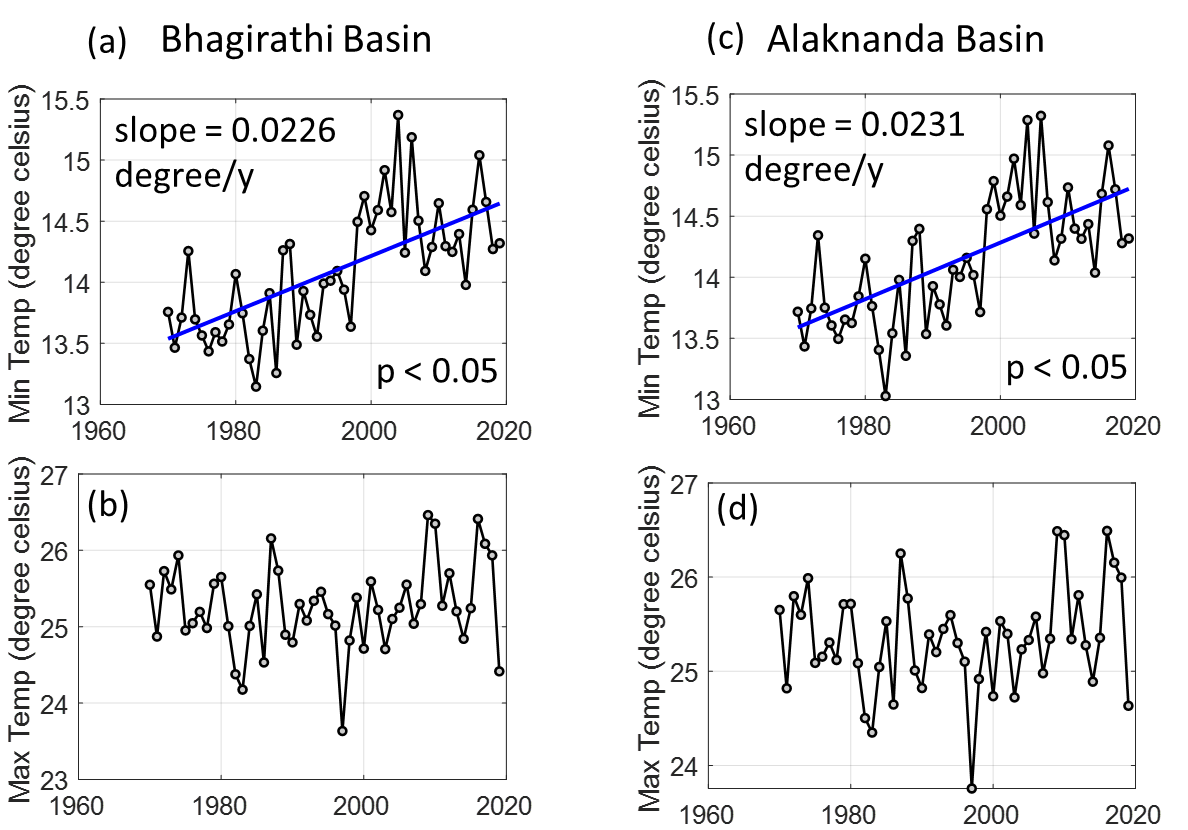
**Figure S1 (a)** mean annual rainfall for period 1970-2019. **(b)** shows the Indian Summer Monsoon (ISM) contributions across different regions in the Upper Ganga Basin (UGB). For rainfall analysis, the IMD 0.25-degree rainfall dataset is used (see Table S1)

**Figure S2** Temperature analyses of the Bhagirathi and Alaknanda River basins. Figures **(a)** and **(c)** show the mean annual minimum temperature variations for the Bhagirathi and Alaknanda basin from 1970 to 2019. There are statistically significant trends at less than 5% significance level present in both the basin. However, the increasing trends detected by the statistical tests are driven by the step-change that occurred between pre-and post-2000, possibly suggesting a shift in the instrumentation. Further, **(b)** and **(d)** show the mean annual maximum tempreture variations for the Bhagirathi and Alaknanda basin from 1970 to 2019. However, there is no statistically significant trends observed at 5% signiifcance level for both the basin. For temperature amalysis, the 1-degree daily temperature data is used (see Table S1)


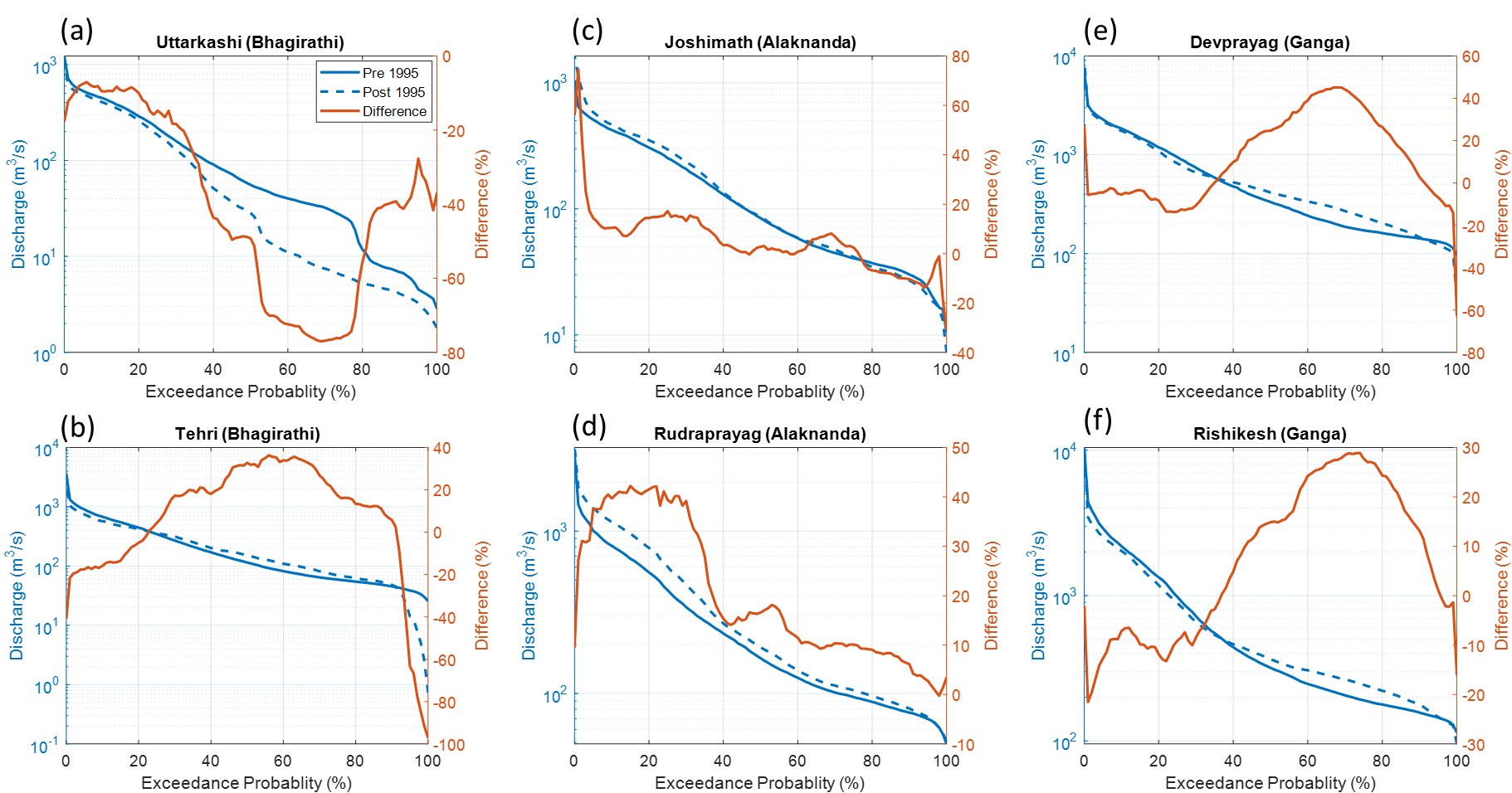


**Figure S3** Post-and pre-1995 flow duration curves (FDCs) and their differences are shown for **(a, b)** the Bhagirathi, **(c, d)** the Alaknanda and **(e, f)** the Ganga basin. For flow duration curve analysis, the daily discharge data is used.


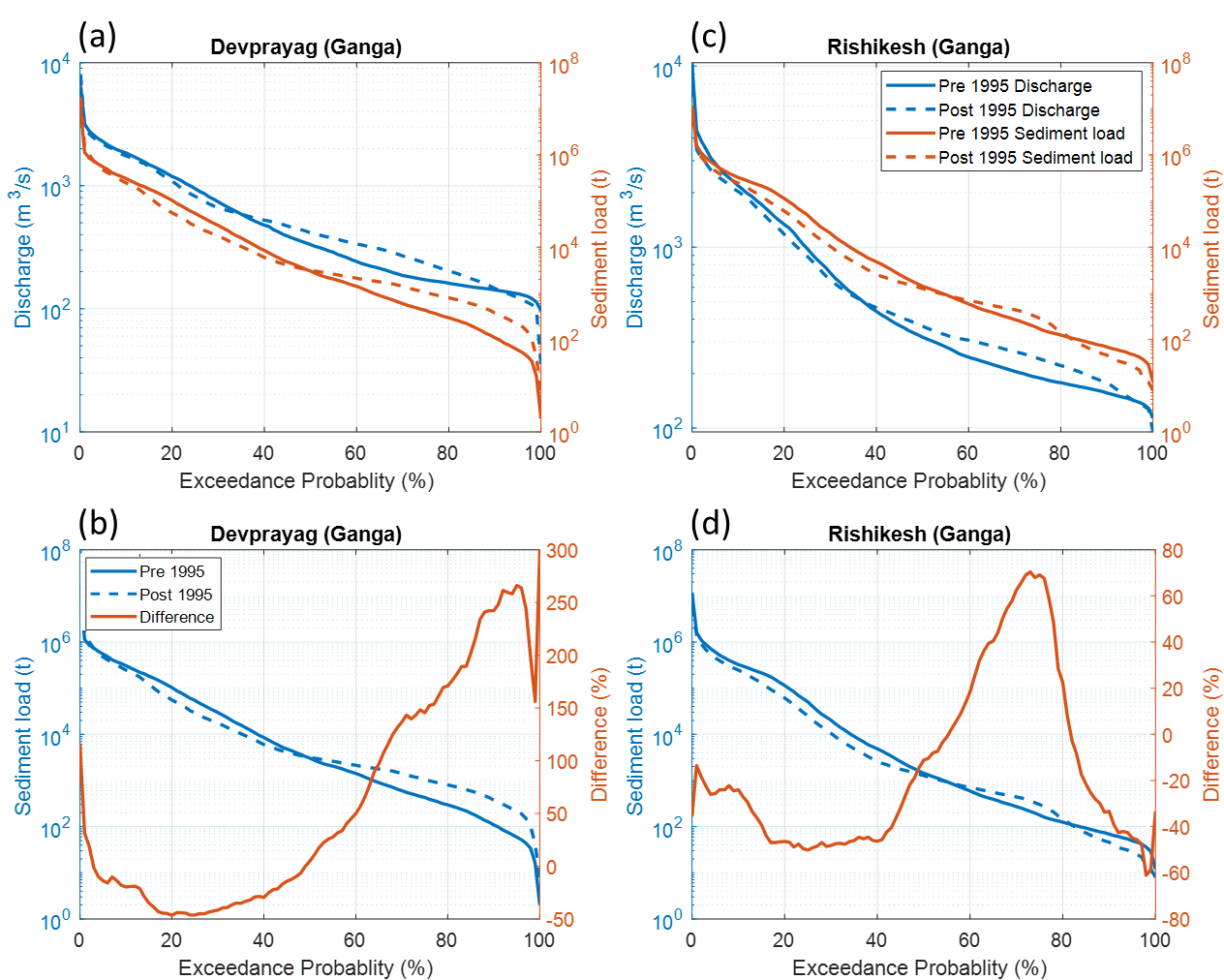


**Figure S4** The comparisons between post-and pre-1995 are shown between the flow duration curves (FDCs) and sediment duration curves (SDCs) at **(a)** Devprayag and **(c)** Rishikesh. The difference between pre-and post-1995 SDCs are also plotted for the **(b)** Devprayag and **(d)** Rishikesh.


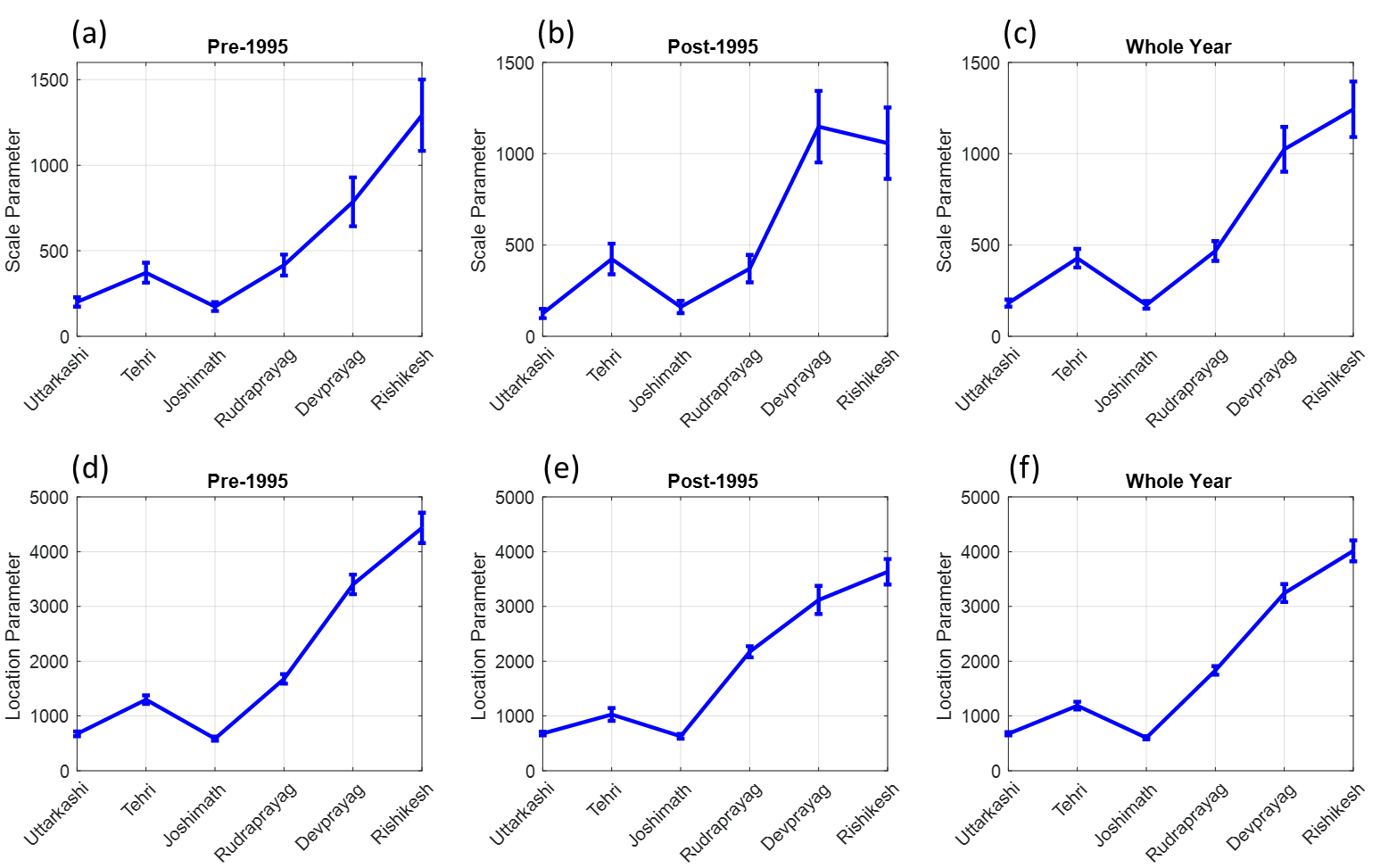


**Figure S5** The estimated scale parameters of the Gumbel distribution for all the stations in the UGB using **(a)** pre-1995, **(b)** post-1995, and **(c)** whole year peak discharge time series. The estimated location parameters of the Gumbel distribution for all the stations in the UGB using **(d)** pre-1995, **(e)** post-1995, and **(f)** whole year peak discharge time series.


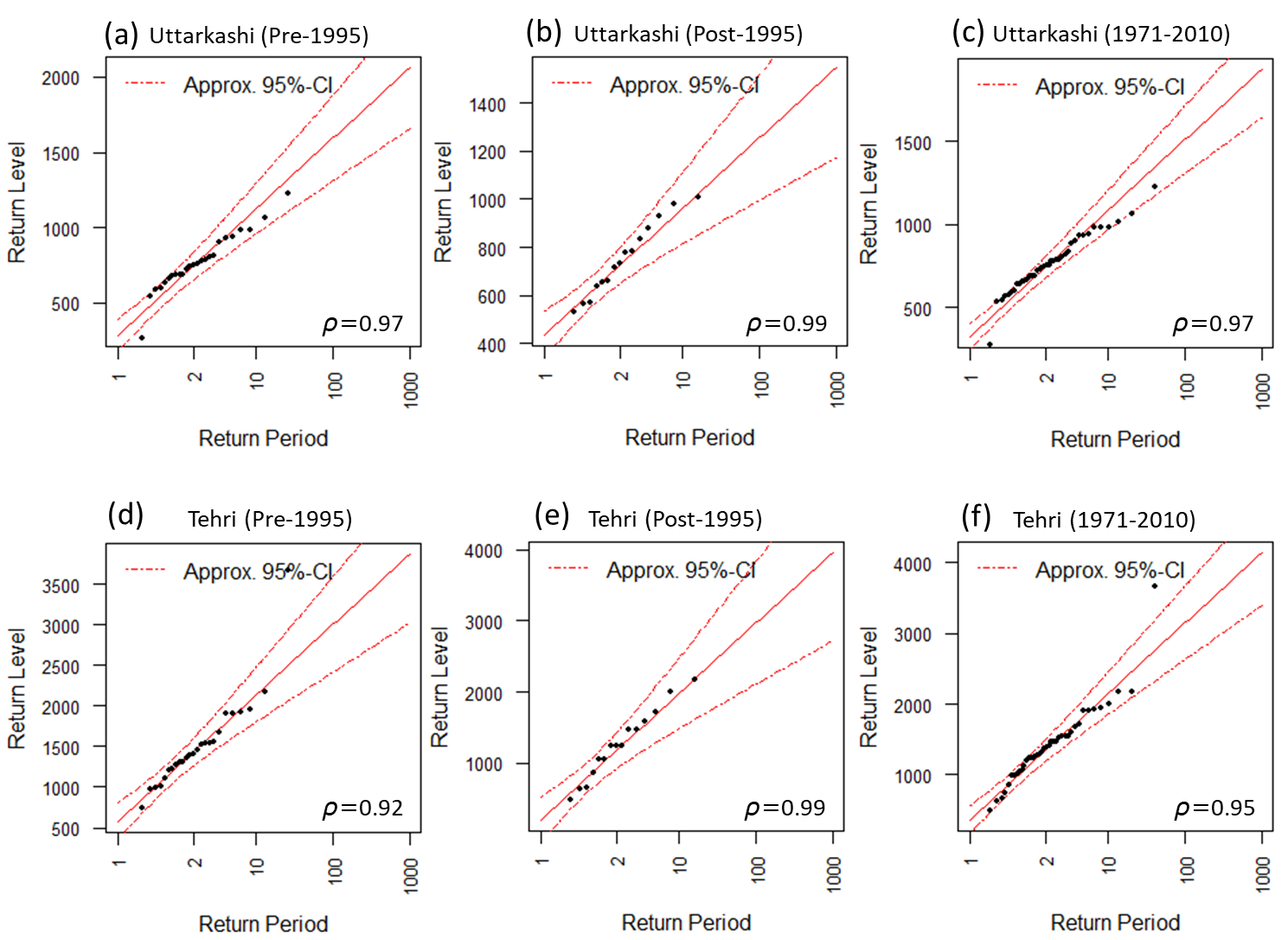


**Figure S6** The return level estimates for Uttarkashi and Tehri gauging stations if the Bhagirathi River basin using **(a, d)** pre-1995, **(b, e)** post-1995, and **(c, f)** whole year (1971-2010) peak discharge time series. The Pearson correlation (ρ) is used to assess the goodness of fit for selected probability distribution in different peak discharge time series.


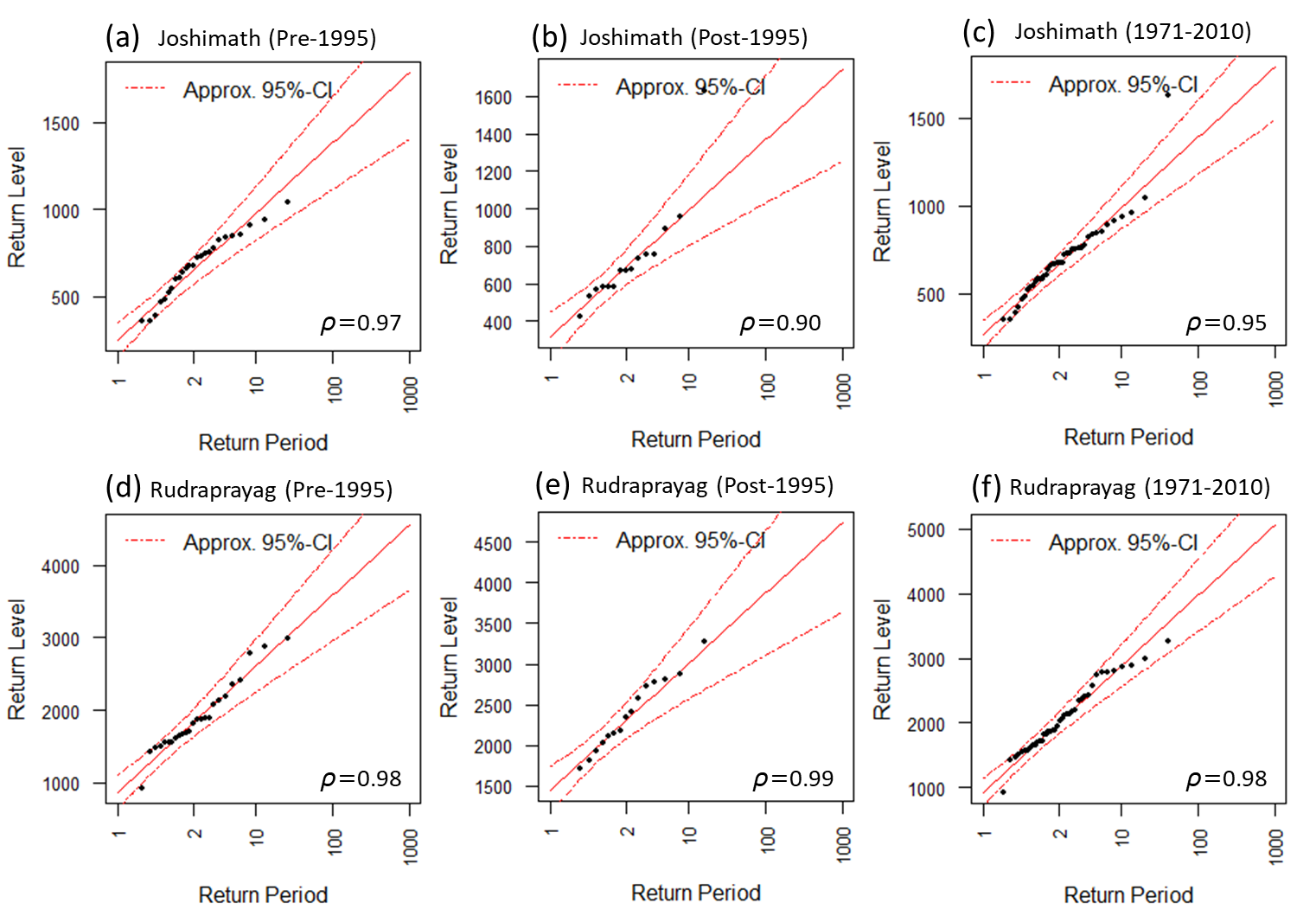


**Figure S7** The return level estimates for Joshimath and Rudraprayag gauging stations of the Alaknanda River basin using **(a, d)** pre-1995, **(b, e)** post-1995, and **(c, f)** whole year (1971-2010) peak discharge time series. The Pearson correlation (ρ) is used to assess the goodness of fit for selected probability distribution in different peak discharge time series.


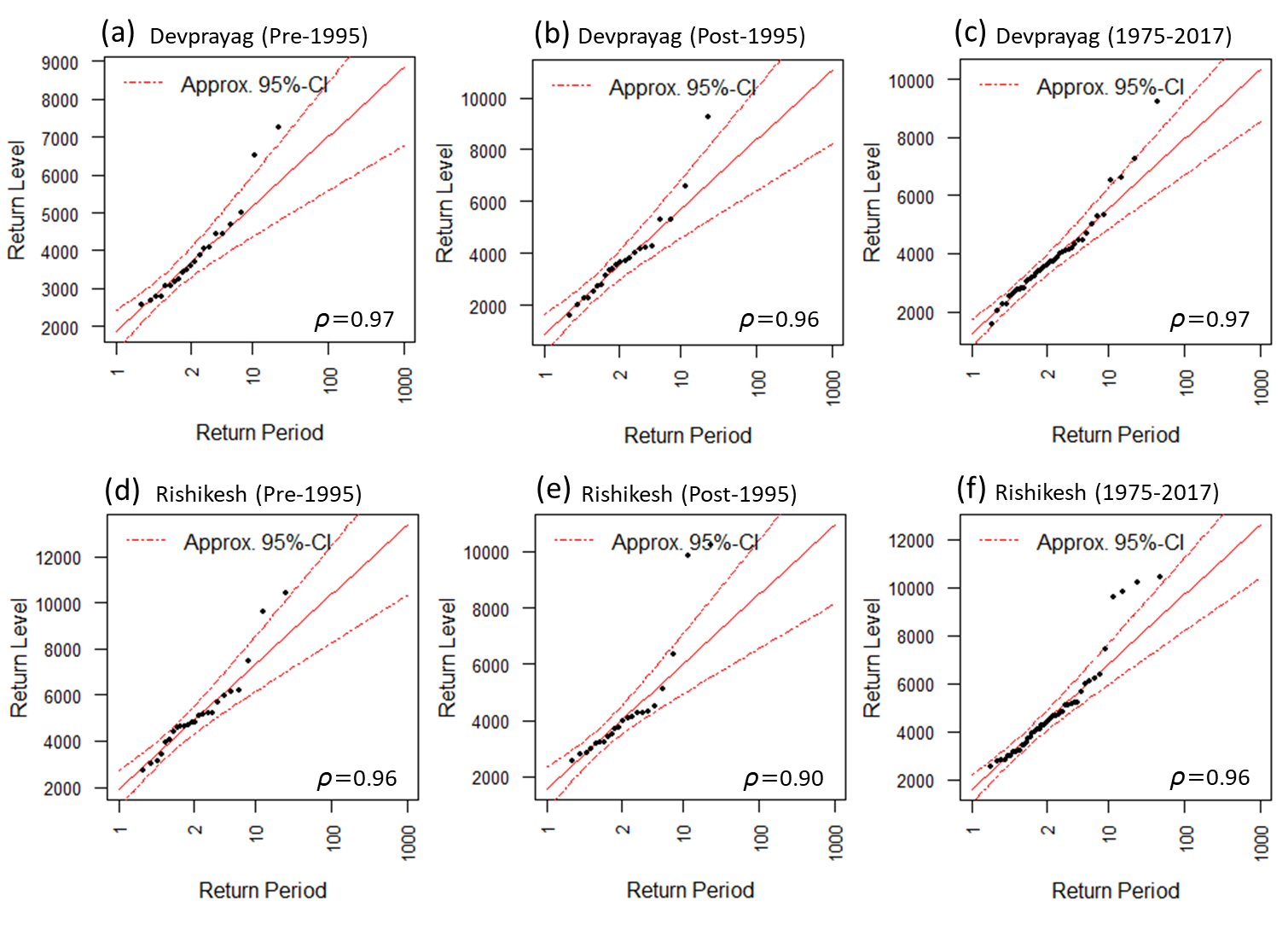


**Figure S8** The return level estimates for Devprayag and Rishikesh gauging stations of the downstream upper Ganga basin using **(a, d)** pre-1995, **(b, e)** post-1995, and **(c, f)** whole year (1975-2015) peak discharge time series. The Pearson correlation (ρ) is used to assess the goodness of fit for selected probability distribution in different peak discharge time series.


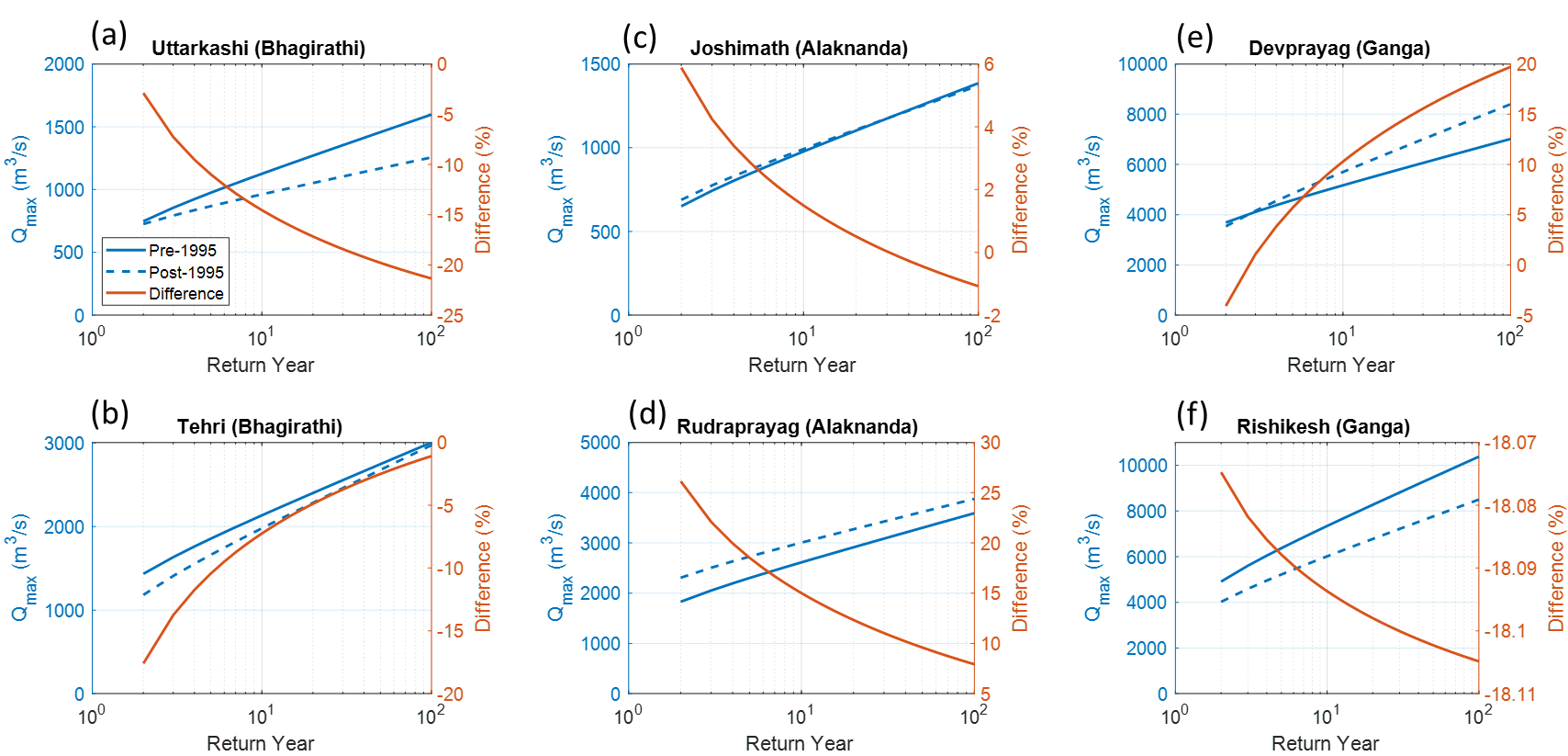


**Figure S9** Pre-and post-1995 extreme flows and their difference are shown for **(a, b)** the Bhagirathi, **(c, d)** the Alaknanda and **(e, f)** the Ganga basin.

**References**

Asthana, A. K. L., & Asthana, H. (2014). Geomorphic control of cloudbursts and flash floods in Himalaya with special reference to Kedarnath area of Uttarakhand, India. *International Journal of Advancement in Earth and Environmental Sciences*, *2*(1), 16-24.

Dimri, A.P., Chevuturi, A., Niyogi, D., Thayyen, R.J., Ray, K., Tripathi, S.N., Pandey, A.K. and Mohanty, U.C. (2017). Cloudbursts in Indian Himalayas: a review. *Earth-Science Reviews*, *168*, pp.1-23.

Gupta, V., Dobhal, D. P., & Vaideswaran, S. C. (2013). August 2012 cloudburst and subsequent flash flood in the Asi Ganga, a tributary of the Bhagirathi river, Garhwal Himalaya, India. *Current science*, 249-253.

Jarvis, A., H.I. Reuter, A. Nelson, E. Guevara, 2008, Hole-filled SRTM for the globe Version 4, available from the CGIAR-CSI SRTM 90m Database ([http://srtm.csi.cgiar.org](http://www.cgiar-csi.org/2010/03/108/uot;http:/srtm.csi.cgiar.org)).

Joshi, V., & Maikhuri, R. K. (1997). Cloudburst: a natural calamity—a case study from Garhwal Himalaya, UP. In *Journal of Indian Building Congress* (Vol. 4, pp. 207-19).

Joshi, V., & Kumar, K. (2006). Extreme rainfall events and associated natural hazards in Alaknanda valley, Indian Himalayan region. *Journal of Mountain Science*, *3*(3), 228-236.

Khanduri, S. U. S. H. I. L., & Sajwan, K. S. (2019). Flash floods in Himalaya with especial reference to Mori tehsil of Uttarakhand, India. *International Journal of Current Research in Multidisciplinary*, *4*(9), 10-18.

Khanduri, S. (2020). Cloudbursts Over Indian Sub-continent of Uttarakhand Himalaya: A Traditional Habitation Input from Bansoli, District-Chamoli, India. *International Journal of Earth Sciences Knowledge and Applications*, *2*(2), 48-63.

Naithani, A. K. (2001). The August, 1998 Okhimath tragedy in Rudraprayag district of Garhwal Himalaya, Uttaranchal, India. *Gaia*, *16*, 145-156.

Naithani, A.K., Kumar, D., Prasad, C. (2002a). The catastrophic landslide of 16 July 2001 in Phata Byung area, Rudraprayag District, Garhwal Himalaya, India. *Current Science,* 82 (25), 921-923.

Naithani, A. K., Joshi, V., & Prasad, C. (2002b). Investigation on the impact of cloudburst in Tehri District, Uttaranchal-31 August 2001. *JOURNAL-GEOLOGICAL SOCIETY OF INDIA*, *60*(5), 573-578.

NIDM (2014). Uttarakhand disaster-2013: lessons learnt and way ahead.

NIDM (2015). Uttarakhand Disaster 2013, National Institute of Disaster Management (Ministry of Home Affairs, Government of India), New Delhi, India.

Pai, D. S., Sridhar, L., Rajeevan, M., Sreejith, O. P., Satbhai, N. S., & Mukhopadhyay, B. (2014). Development of a new high spatial resolution (0.25× 0.25) long period (1901–2010) daily gridded rainfall data set over India and its comparison with existing data sets over the region. *Mausam*, *65*(1), 1-18.

Prakash, S. (2015). *A study on flash floods and landslides disaster on 3rd August 2012 along Bhagirathi Valley in Uttarkashi District, Uttarakhand*. World Centre of Excellence on Landslide Disaster Reduction, National Institute of Disaster Management, Ministry of Home Affairs, Government of India.

Sati, V. P. (2007). Environmental impacts of debris flows-A case study of the two debris-flow zones in the Garhwal Himalaya. *Debris-Flow Hazards Mitigation: Mechanics, Prediction, and Assessment, Chen & Major, eds*, 715-723.

Sah, M. P., Asthana, A. K. L., & Rawat, B. S. (2003). Cloud burst of August 10, 2002 and related landslides and debris flows around Budha Kedar (Thati Kathur) in Balganga valley, district Tehri. *Himalayan Geology*, *24*(2), 87-101.

Srivastava, A. K., Rajeevan, M., & Kshirsagar, S. R. (2009). Development of a high resolution daily gridded temperature data set (1969–2005) for the Indian region. *Atmospheric Science Letters*, *10*(4), 249-254.
